# Fish the ChIPs: a pipeline for automated genomic annotation of ChIP-Seq data

**DOI:** 10.1186/1745-6150-6-51

**Published:** 2011-10-06

**Authors:** Iros Barozzi, Alberto Termanini, Saverio Minucci, Gioacchino Natoli

**Affiliations:** 1 Department of Experimental Oncology, European Institute of Oncology (IEO), IFOM-IEO Campus, Via Adamello 16, Milan, Italy; 2 Universita' degli Studi di Modena e Reggio Emilia, Dipartimento di Scienze Biomediche, Via Giuseppe Campi 287, Modena, Italy; 3 Universita' degli Studi di Milano, Dipartimento di Scienze Biomolecolari e Biotecnologie - Via Celoria 26, Milano, Italy

## Abstract

**Background:**

High-throughput sequencing is generating massive amounts of data at a pace that largely exceeds the throughput of data analysis routines. Here we introduce Fish the ChIPs (FC), a computational pipeline aimed at a broad public of users and designed to perform complete ChIP-Seq data analysis of an unlimited number of samples, thus increasing throughput, reproducibility and saving time.

**Results:**

Starting from short read sequences, FC performs the following steps: 1) quality controls, 2) alignment to a reference genome, 3) peak calling, 4) genomic annotation, 5) generation of raw signal tracks for visualization on the UCSC and IGV genome browsers. FC exploits some of the fastest and most effective tools today available. Installation on a Mac platform requires very basic computational skills while configuration and usage are supported by a user-friendly graphic user interface. Alternatively, FC can be compiled from the source code on any Unix machine and then run with the possibility of customizing each single parameter through a simple configuration text file that can be generated using a dedicated user-friendly web-form. Considering the execution time, FC can be run on a desktop machine, even though the use of a computer cluster is recommended for analyses of large batches of data. FC is perfectly suited to work with data coming from Illumina Solexa Genome Analyzers or ABI SOLiD and its usage can potentially be extended to any sequencing platform.

**Conclusions:**

Compared to existing tools, FC has two main advantages that make it suitable for a broad range of users. First of all, it can be installed and run by wet biologists on a Mac machine. Besides it can handle an unlimited number of samples, being convenient for large analyses. In this context, computational biologists can increase reproducibility of their ChIP-Seq data analyses while saving time for downstream analyses.

**Reviewers:**

This article was reviewed by Gavin Huttley, George Shpakovski and Sarah Teichmann.

## Background

Chromatin-immunoprecipitation followed by massively-parallel sequencing (ChIP-Seq) has become the most popular and effective technique to investigate chromatin states and distribution of transcription factors at the genomic level [1,2].

The throughput of next generation sequencing is growing at a pace that largely exceeds the evolution of routine pipelines for downstream data analyses [3]. While there have been constant improvements regarding the alignment and the peak calling steps in ChIP-Seq experiments, comparatively little efforts have been made in order to automate the entire process and reduce any possible impact of manual data processing on the final results. In this context, our aim is to define a standardized approach to analyze and validate ChIP-Seq data. Fish the ChIPs (FC) is a novel computational pipeline able to perform a complete first-level ChIP-Seq analysis, from raw short sequence reads to complete genomic annotation of the enriched regions. In order to allow processing of big batches of samples in an automated and reproducible fashion, FC gathers and coordinates some of the fastest and most effective tools available in the literature. Besides, biologists can install it on a Mac desktop machine and run it through a graphic user interface. Every single step is linked to the following one by a collection of *ad hoc *custom scripts. These scripts also provide original functionalities such as the creation of pie charts representing the genomic distribution of the peaks, as well as histograms of their distribution around transcriptional start sites (TSS) and 3' untranslated regions (3' UTR). Besides the computational pipeline, we suggest a simple and effective methodology to validate the resulting enriched regions. The pipeline has been developed for data coming from Solexa Illumina Genome Analyzers and ABI SOLiD but can be run with data coming from other platforms. Every single tool that has been included in FC has been published or is freely available. The custom scripts are provided under a GNU General Public License. We also aim at keeping the tool updated as soon as more comprehensive and/or more performing tools will be available.

## Results and Discussion

### The steps of the pipeline

After conversion of either srf or sra raw files to fastq (using srf2fastq - a tool part of the Staden package [4] - in case of srf or the SRA Toolkit [5] in case of sra), quality control reports are generated using FastQC [6]. Reads are then aligned to a reference genome using Bowtie [7]. Bowtie has been proven to be the fastest among the open source short-reads aligners [8].

For the peak calling procedure we chose MACS [9]. In a recent comparison of five extensively used peak-finding programs [10], MACS has been demonstrated to have the best performance over manually curated datasets. Moreover, it is able to model the shift size of the tags using the strand information from the most enriched genomic regions, defined through the mfold parameter. Compared to previous releases, MACS 1.4 is able to explore the space of this parameter in order to find the one most suitable to the dataset considered. It also creates genomic wiggles files for the UCSC genome browser [11].

Gene Interval Notator (GIN) [12] is a simple tool written for the annotation of genomic regions over tabular datasets extracted from the UCSC genome browser. The genomic list of features provided to the GIN for the annotation must be in UCSC genome browser tabular format and can be of any type (e.g. protein coding genes, mRNAs, ncRNAs). We chose GIN over other existing tools [13,14] because it is available as a standalone script and returns a simple and clear output - namely each peak assigned to a single genomic element (promoter, exon, intron or intergenic). After annotation, tables coming from GIN are used as input for a set of custom scripts (merge, PIES_PEAKS_GD, TSS_3UTR_dist) aimed at generating a tabular annotation of the enriched regions as well as annotation plots (pies showing genomic distributions and density plots of the peaks surrounding TSS and 3'UTR).

Recent advancements in the data formats supported by the UCSC genome browser [11] are heading towards a switch from wiggle to BigWig format. In this context we include wigToBigWig, a freely available tool aimed to convert to BigWig the wiggle files generated by MACS. FC also exploits the IGVTools [15] in order to generate tdf tracks, which can be visualized on the Integrative Genomics Viewer (IGV) [15].

**Figure 1:**
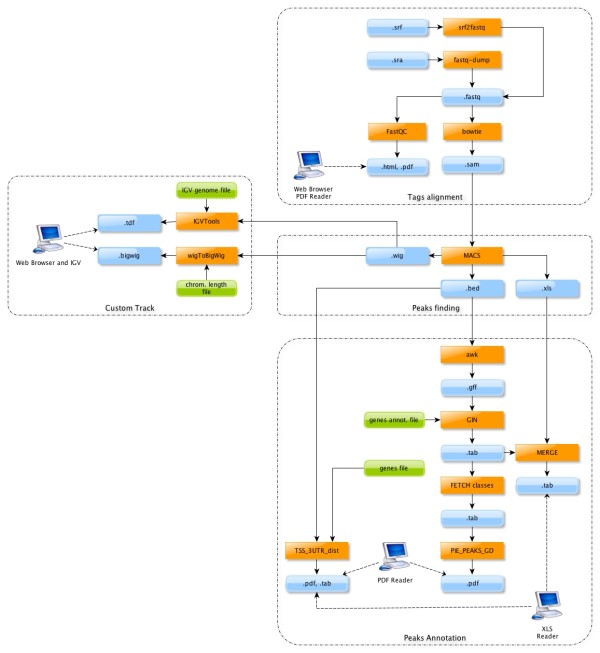
**Schematic representation of the pipeline**. Schematic representation of the pipeline.

The flowchart in Figure 1 encompasses all the steps described above.

### Installing and configuring the pipeline

The installation procedure of the Mac implementation of FC is completely automated and includes all the required external components. After installation, the next and only step for a Mac user is to run the graphic interface and specify the tag size and the species of interest along with the samples and the different comparisons to be performed among them. All the other parameters are set to default even though the user can customize any of them. If the user builds FC from binaries, the pipeline must be run from the command line. In this case, she/he has to create a suitable configuration file that specifies all the parameters. The user can generate it either using a dedicated public web-application http://bio.ifom-ieo-campus.it/ftc/ or following the instructions provided in the user guide http://bio.ifom-ieo-campus.it/ftc/manual.html. In order to run the pipeline using raw data generated with sequencing technologies other than Illumina Solexa Genome Analyzers, the user has to apply only very small changes in the parameters. First of all, technologies like SOLiD work in color-space. Bowtie supports it, so we give the possibility to the user to download a color-space index for relevant species during the installation process.

### Comparison with other tools available in the literature

Compared to other ChIP-Seq pipelines available in the literature [16-18], FC encompasses all the single steps needed for a complete analysis from raw data to genomic annotations. Differently from available tools, FC has two main advantages. Namely, it provides a graphic user interface and it can handle an unlimited number of pairwise comparisons among an unlimited number of samples. Besides, the user can specify one or more raw files for each sample. In this case, FC pulls together reads coming from different replicates. See Table 1 for a point-to-point comparison of FC to the other tools available in the literature. Compared to [16,18] FC is not performing any motif discovery step. We chose to not include it because such a follow-up step is less prone to be automated and each single dataset analyzed usually needs an independent step to define suitable parameters.

**Table 1:** Comparison among FC and similar tools available in the literature

	FC	**CisGenome **[16]	**Sole-Search **[17]	[18]
**Raw conversion**	Yes	No	Yes	No
**SOLiD data support**	Yes	Not explicit	Not explicit	Not explicit
**Quality control**	Yes	No	No	No
**Alignment**	Yes (Bowtie)	No	Yes	No
**Peak Calling**	Yes (MACS)	Yes (SeqPeak)	Yes	Yes (PICS)
**Annotation**	Yes	Yes	Yes	Yes
**Wiggle file**	Yes	Yes	Yes	No
**bigWig file**	Yes	No	No	No
**tdf file**	Yes	No	No	No
**Motif Analysis**	No	Yes	No	Yes
**Samples comparison**	Unlimited	One pair	One pair	One pair
**Handle replicates**	Yes	No	Yes	No
**Single package**	Yes	Yes	Yes	No

### A strategy for ChIP-Seq validation

The validation step is aimed at demonstrating the reproducibility of the result. Therefore, it is usually performed on biologically independent samples through a different technique able to quantify the enrichment (e.g. quantitative-PCR). An effective procedure is to rank the dataset of called peaks from the most to the least significant one (based on the score MACS assigns to each enriched region), and then split it in progressive groups. Every different bin will represent a homogeneous group of regions in terms of enrichment or statistical significance. For each group a suitable number of regions will then be randomly chosen for the validation. Given the percentage of validated regions in each group, the user will then set the appropriate threshold [19].

### Running time

We tested FC on two ChIP-Seq experiments in mouse cells (GEO accession numbers: GSM487450 and GSM487448, ~13 millions and ~14 millions of raw short reads) against the correspondent input DNA (GSM487453) using four Intel(R) Xeon(R) E7450 core CPU (2.40 GHz) and 4 GB RAM. Results are summarized in Table 2. Given a comparable number of short reads, the time consumed by each step is roughly the same in both cases except for the annotation of the peaks. This is due to the fact the number of peaks called for sample GSM487448 largely exceeds sample GSM487450. At least for datasets with this sequencing depth, we demonstrate the feasibility of running FC on a desktop computer.

**Table 2:** Running time

	GSM487450	GSM487448
**Raw conversion**	5	7
**Quality control**	6	5
**Alignment**	40	43
**Peak Calling**	30	38
**Genomic annotation**	64	1
**bigwig/tdf conversion**	3	6
**Total**	147	100

FC running time in minutes using two different ChIP experiments (GEO accession numbers: GSM487450 and GSM487448) against the correspondent input DNA (GSM487453). Test were run on a machine with four Intel(R) Xeon(R) E7450 core CPU (2.40 GHz) and 4 GB RAM.

## Conclusions

FC provides a useful framework to perform ChIP-Seq analyses in a fast and completely automated manner. FC is a very flexible tool and is then suitable for a broad public of users. To this end, we provide Mac binaries and a graphic user interface besides source code. This way, also wet biologists with very basic computational skills can perform ChIP-Seq analysis starting from the raw data. The installation procedure is completely automated and includes a step to download the indexes of the genomes of interest required for the reads alignment step. At the same time, computational biologists can exploit FC to analyze large batches of data saving time and reducing errors, still keeping under control each single parameter of the analysis.

## Methods

### Overview of the tools used during the pipeline development process

We developed the FC main code in C++ (compiled with g++), while single scripts called by the pipeline are either written in C++, Python, Perl or R. The website through which the user can generate configuration files is written in PHP. yEd Graph Editor [20] was used to draw the pipeline schema.

## Competing interests

The authors declare that they have no competing interests.

## Authors' contributions

IB and AT designed and packaged the pipeline. IB wrote the paper. GN and SM gave substantial contribution in drafting and revising the paper. All authors read and approved the final manuscript.

## Reviewers' comments

### Reviewer's report 1

Gavin Huttley, John Curtin School of Medical Research, Australia

Fish the Chips (FC) is a software package that aims to simplify the computational process of peak prediction from ChIP-Seq data with an OSX front end. It is also available, in non-GUI form, for standard Unix environments.

I do not recommend users on OSX or Unix install this tool. If a reader does install this software they are advised to backup their computer first.

**Author's response: ***The package is not affecting any software previously installed on the target machine. The reviewer raised an issue about the Python installation (see below), which it has been solved. We would like to point out that the following comments could only be related to the Mac installation and not to the Unix one. As described in the manual, the general Unix installation is not installing any Python or R on the user machine*.

My principal concern is FC seems likely to generate conflicts with a users other installed software. Looking at the install log (page 4 of supplementary material) indicates the installation of Python 2.6.6. On OSX 10.6, Apple provides Python 2.6.1 as a default, implying that this installed package is 'Mac Python' from python.org. When Python comes bundled with OSX, why does a full version of Python need to be installed on users machines at all?

If this installation process modifies the users PATH environment to point to this new Python, then a user would no longer be able to seamlessly use other Python packages a user had previously installed.

**Author's response: ***MACS (Model-based analysis of ChIP-Seq (MACS). Zhang et al.*, *Genome Biol 2008) authors recommend Python 2.6.6 or higher for MACS proper execution. This is the reason the user must be sure it is installed on the target machine before running FC. We understand the reviewer's concern, and we overcame the issue with two solutions. First of all, we provide two installers, one specific for Lion and one for Snow Leopard. Lion has already Python 2.7.1 installed on it so there is no need to install any other Python release. We also changed the Snow Leopard version of the installer so that Python 2.6.6 will not overwrite any existing Python on the target machine. MACS will exploit this particular Python installation while the Python invoked by default will be unchanged*.

A similar overwriting of the users R compute environment also seems possible. For these reasons I chose not to install and try this package.

**Author's response: ***The R installation performed by FC will not overwrite any previously installed version. We also added a check so that R is installed only in case any previous installation is not found*.

The issues I raise above are not insurmountable. From the described implementation details, the full capabilities of this software could be delivered using Apple's own Python framework plus bundling up the other scripts. The whole package should be installable as a drag-and-drop application with all dependencies included, leaving the users Unix environment untouched. For instance one can use Apple's own Python.framework, instead of that from Python.org, and include any custom scripts within the Applications own Resource folder. The application can then modify the PATH and PYTHONPATH environment variables during execution within it's own process before spawning subprocesses. One seeming blocker to this strategy would be R, but this too can be addressed. R is being used solely to produce simple graphics (pie charts and histograms). At least one solution is to the obtain these capabilities from other software such as the Python matplotlib library http://matplotlib.sourceforge.net/. Thus, the dependency on R can be eliminated by including matplotlib within the application bundle.

**Author's response: ***Matplotlib requires *Python.org, *so it is not working under Apple's own Python.framework. That means we should have installed Python in any case*.

With such a true OSX app bundle, the un-install process would be greatly simplified --drag to trash. At present, I predict un-install is likely to be complicated even for an expert Unix user.

For non-OSX users the functionality seems minimal and of limited use. For instance, it is not obvious to me from the current description at what point, if at all, adapter sequences or low quality bases are removed. This is essential for any analyses since sequences with adapters frequently fail to align.

**Author's response: ***In the first step of the analysis, we added a flag for removing low quality reads*.

The rate of development of individual applications for the analysis of ChIP-seq data makes it likely that some of the tools connected by this application will be superseded rapidly. Given this and the limited function set of FC I suggest bioinformaticians are better off writing their own controllers.

**Author's response: ***As we explicitly added in the text, we aim at keeping the pipeline updated as soon as more comprehensive or more performing tools will be released*.

### Reviewer's report 2

George Shpakovski, Shemyakin-Ovchinnikov Institute of Bioorganic Chemistry, Russian Academy of Sciences, Russian Federation The authors have developed a new computational pipeline called Fish the ChIPs (FC) which is specifically designed to perform complete ChIP-Seq (chromatin-immunoprecipitation followed by multi-parallel sequencing) data analysis of an unlimited number of samples. The FC was adapted to work with data coming from Illumina Solexa Genome Analyzers or ABI SOLiD, but the pipeline usage can be potentially extended to other sequencing platforms. Comparison of the FC with similar tools available from the literature (Table 1) indicate that FC is one of the most effective tools for ChIP-Seq data analysis today available and could probably became a popular technique among researches investigating chromatin states and transcription factors' distribution at the genomic level. Although the authors tried to make the FC installation procedure automated as much as possible, it would be helpful if they could provide short instructions for the pipeline installation not only for Mac users but also for scientists using IBM-compatible computers in their research.

**Author's response: ***It would be very difficult for wet biologists to install every component of FC on a machine running Windows OS without an automatic installer. For this reason, we are not providing any instruction for it in the manual. Nevertheless, we could provide a package in the future*.

### Reviewer's report 3

Sarah Teichmann, MRC Laboratory of Molecular Biology, United Kingdom

This pipeline unifies several existing programs (mapping, peak-picking) and scripts (file conversions etc). There are already several similar published packages, so it is a bit disappointing that this one doesn't try to provide a novel angle on this data processing task.

**Author's response: ***We do not agree. We provide a Mac OS X package that is easy to install and a graphical user interface that can be used by wet biologist to analyze their own ChIP-seq data. Besides, differently from any other tool of the same kind available in the literature, FC is able to manage an unlimited number of samples and comparison, allowing computational biologists to increase the reproducibility of their results while saving time*.

Nevertheless, there are a few new ideas in it, such as providing a set of regions suitable for qPCR validation. This section is not very well presented in the manuscript at the moment: it took me quite a while to understand that the package is "Identifying regions for validation" rather than doing "ChIP-seq validation" - the current title.

**Author's response: ***We changed the title of the paragraph to "A strategy for ChIP-seq validation"*.

A further new aspect is the incorporation of SOLID data into FC, which the other packages do not offer to my knowledge. If this is correct, it would be worth including it into Table 1.

In terms of comparing the existing packages, it would be helpful to have a comparison between them (FC vs refs 11-13) in terms of both a transcription factor and epigenetic ChIp-seq dataset. What are the differences in peaks (epigenetic) and target genes (transcription factor) that are assigned by the different packages? For the assignment of target genes to peaks, FC simply takes the closest gene or even TSS. It is known that this introduces a severe bias, which approaches such as GREAT (Bejerano group) try to circumvent. ChIPpeakAnno (Green and co-workers) provides a higher-level analysis of the patterns of peaks relative to genes, GO categories etc. These methods should at least be cited, if not included in FC itself.

**Author's response: ***We agree about GREAT and we plan to include GREAT annotation beside the canonical "best-hit" annotation in a future version of FC. Compared to ChIPpeakAnno, GIN is able to assign each peak to a single genomic element (promoter, exon, intron or intergenic) in a single run, generating a very simple and clear output. For this reason we chose GIN over ChIPpeakAnno*.
